# A high-throughput method for quantifying alleles and haplotypes of the malaria vaccine candidate *Plasmodium falciparum *merozoite surface protein-1 19 kDa

**DOI:** 10.1186/1475-2875-5-31

**Published:** 2006-04-20

**Authors:** Shannon L Takala, David L Smith, O Colin Stine, Drissa Coulibaly, Mahamadou A Thera, Ogobara K Doumbo, Christopher V Plowe

**Affiliations:** 1 Center for Vaccine Development, University of Maryland School of Medicine, 685 West Baltimore Street, HSF1-480, Baltimore, Maryland 21201, USA; 2 Malaria Research and Training Center, University of Bamako, BP 1805, Bamako, Mali; 3 Fogarty International Center, National Institutes of Health, 16 Center Drive, Room 202, Bethesda, Maryland 20892, USA; 4 Department of Epidemiology and Preventive Medicine, 660 West Redwood Street, University of Maryland School of Medicine, Baltimore, Maryland 21201, USA

## Abstract

**Background:**

Malaria vaccine efficacy may be compromised if the frequency of non-target alleles increases following vaccination with a genetically polymorphic target. Methods are needed to monitor genetic diversity in polymorphic vaccine antigens, but determining which genetic variants of such antigens are present in infected individuals is complicated by the frequent occurrence of mixed infections.

**Methods:**

Pyrosequencing was used to determine allele frequencies at each of six single nucleotide polymorphisms in the *Plasmodium falciparum *blood-stage vaccine antigen merozoite surface protein 1 19 kDa (MSP-1_19_) in field samples from a vaccine-testing site in Mali. Mixtures of MSP-1_19 _clones were created to validate a haplotype-estimating algorithm that uses maximum likelihood methods to determine the most probable combination of haplotypes given the allele frequencies for an infection and the haplotypes known to be circulating in the population.

**Results:**

Fourteen unique MSP-1_19 _haplotypes were identified among 351 genotyped infections. After adjustment to a standard curve, Pyrosequencing provided accurate and precise estimates of allele frequencies in mixed infections. The haplotype-estimating algorithm provided accurate estimates of haplotypes in mixed infections containing up to three haplotypes. Based on the MSP-1_19 _locus, approximately 90% of the 351 infections contained two or fewer haplotypes.

**Conclusion:**

Pyrosequencing in conjunction with a haplotype-estimating algorithm provides accurate estimates of haplotypes present in infections with up to 3 haplotypes, and can be used to monitor genetic diversity in parasite populations prior to and following introduction of MSP-1-based malaria vaccines.

## Background

Malaria remains a major cause of disease and death in tropical regions. A malaria vaccine could contribute to malaria control, but as with other pathogens (e.g. HIV, *Streptococcus pneumoniae*, and influenza virus), malaria vaccine development is complicated by genetic diversity in vaccine antigens. Most malaria vaccine antigens have a high rate of non-synonymous amino acid substitutions and continue to evolve under selection from the immune system [1-3]. If immunity conferred by a subunit vaccine is allele-specific, then vaccination could lead to an increased frequency of alleles not targeted by the vaccine. Such changes in the parasite population could compromise vaccine efficacy. It is therefore important to understand the genetic diversity in polymorphic antigens in endemic populations before and after the introduction of a malaria vaccine, including the prevalence of different genetic variants and their natural dynamics, and to measure allele-specific protective efficacy in clinical trials of malaria vaccines.

Merozoite Surface Protein 1 is a leading malaria vaccine candidate antigen. It is the most abundant protein on the surface of the merozoite and is synthesized as a 195 kDa precursor. After undergoing proteolytic cleavage, only the c-terminal 19 kDa remains on the surface of the merozoite as it enters the erythrocyte [4]. The 19 kDa fragment contains two epidermal growth factor (EGF)-like domains, which are thought to have an important function in erythrocyte invasion [5]. Antibodies to this region can block erythrocyte invasion in vitro [4] and are associated with protection from clinical malaria in field studies [6-8]. The sequence of MSP-1_19 _is highly conserved [9], which, along with its putative critical function, make it an attractive vaccine target. However, this region has six non-synonymous single nucleotide polymorphisms (SNPs) at amino acid positions 1644, 1691, 1699, 1700, 1701, and 1716 [9-12], which result in expression of different amino acids at those sites (e.g. EKSNGL, QKSNGF, ETSSRL, etc.). It is not known whether or how this polymorphism affects immunity.

Determining which genetic variants of polymorphic malaria antigens are present in infected individuals is complicated by the frequent occurrence of mixed infections. When region of interest is amplified using polymerase chain reaction (PCR), the product and subsequent sequence generated by direct DNA sequencing represents the pool of all parasite types present in that infection. Consequently, it is difficult to distinguish which nucleotides reside together on one parasite (i.e. haplotypes). Haplotypes can be identified using PCR cloning [11,13], since each clone contains a single copy of the amplified region of interest; however, cloning is time consuming, expensive, and not all sequences clone with equal efficiency.

Pyrosequencing™ (Biotage, Charlottesville, VA) is a real-time sequencing method that detects release of pyrophosphate during nucleotide incorporation by an enzyme cascade that generates light proportional to the amount of nucleotide incorporated. This technique allows sequencing of short stretches of nucleotides (10–20 bp) surrounding known polymorphisms without sequencing the rest of the conserved sequence. Pyrosequencing software can quantify the proportion of each alternative nucleotide at each SNP site based on relative peak heights. This method has been shown to provide accurate and precise measurements of allele frequencies in pools of human DNA [14,15] and of the degree of DNA methylation [16].

If allele frequencies at each polymorphic site in a candidate antigen can be determined, and it is known which unique haplotypes are circulating in the population, then a mathematical model can be developed to estimate which haplotypes are present in mixed infections. In this study, Pyrosequencing was used to determine allele frequencies at each of the six SNPs in MSP-1_19_, and an algorithm was developed to reconstruct the frequency of MSP-1_19 _haplotypes in mixed malaria infections. This method will provide a time- and cost-effective alternative to PCR cloning for monitoring parasite populations before and after vaccine introduction.

## Methods

### Samples and DNA extraction

All samples used in this study were collected at a malaria vaccine-testing site in Bandiagara, Mali. DNA was extracted from 3 MM Whatman (Whatman Inc., Clifton, NJ) filter paper blood samples using a QIAamp DNA Mini Kit (Qiagen, Valencia, CA). PCR followed by direct sequencing was used to screen 55 samples collected from children participating in a case-control study of severe malaria [17]. PCR followed by Pyrosequencing was used to screen 296 samples collected from children and young adults participating in a malaria incidence study [18]. Both the case-control study and the malaria incidence study were conducted during the years 1999–2001, and were approved by Institutional Review Boards of the University of Bamako Faculty of Medicine and the University of Maryland Baltimore. Samples with sequences consistent with the presence of unique  MSP-1_19_ haplotypes were identified and one representative of each haplotype underwent PCR cloning.

### PCR

PCR primers were designed, using Pyrosequencing Assay Design Software version 1.0.6 (Biotage, Charlottesville, VA), to amplify 272 bp of MSP-1_19 _containing the six SNPs of interest (forward: 5'-CAATGCGTAAAAAAACAATGTCC-3', reverse: 5'-TTAGAGGAACTGCAGAAAATACCA-3'). The reverse primer contains a 5' biotin label. Each 50 μl PCR contained 33.35 μl sterile distilled water, 5 μl 10× PCR buffer (Qiagen, Valencia, CA), 4 μl MgCl_2 _(25 mM), 0.4 μl DNTPs (100 mM with nucleotides mixed in equal proportions), 1 μl each of forward and reverse primer (5 μM), 0.25 μl HotStarTaq polymerase (Qiagen, Valencia, CA), and 5 μl template DNA. Cycling conditions were as follows: 95°C for 15 minutes; 94° for 30 seconds, 65° for 30 seconds, and 72° for 30 seconds for 10 touchdown cycles -0.5°/cycle; 94° for 30 seconds, 60° for 30 seconds, and 72° for 30 seconds for 35 cycles; and a final extension at 72° for 10 minutes. PCR products were visualized on 1.5% agarose gels.

### Pyrosequencing

For each Pyrosequencing reaction, 5–10 μl of each biotinylated PCR product (depending on product yield) was aliquotted into the wells of a 96-well plate. To bind the products to sepharose beads, 70 μl of binding reaction mix was added to each well. The binding reaction mix consists of 40 μl Binding Buffer (Biotage, Charlottesville, VA), 28 μl high purity water, and 2 μl Streptavidin-Sepharose™ beads (Amersham Biosciences, Piscataway NJ). The binding reaction mix and PCR products were mixed at 1400 rpm at room temperature for at least five minutes.

Four Pyrosequencing reactions are required to genotype the six SNPs in MSP-1_19_. Table 1 shows the sequence of the primers for each Pyrosequencing reaction. Primers were designed using Pyrosequencing Assay Design Software version 1.0.6 (Biotage, Charlottesville, VA). Each Pyrosequencing primer was diluted to 0.417 μM in Annealing Buffer (Biotage, Charlottesville, VA), and 12 μl of the annealing mix (including Pyrosequencing primer) was added to each well of a PSQ™ HS 96-well plate, resulting in 5 pmol of Pyrosequencing primer per well. Negative controls (i.e. Pyrosequencing primer only and biotinylated primer and Pyrosequencing primer without template) were included on each plate to confirm that background signal was negligible.

**Table 1:** Pyrosequencing primers used to genotype polymorphisms in MSP-1_19_.

**SNP**			**Primer**
**Location**	**Nucleotides**	**Amino Acids**	
1644	G/C	E/Q	5'-GCGTAAAAAAACAATGTC-3'
1691	A/C	K/T	5'-GTGATGCAGATGCCA-3'
1699^a^	G/A	S/N	5'-CCGAAGAAGATTCAGGTA-3'
1700^a^	G/A	S/N	
1701^a^	G/A	G/R	
1716^b^	C/T	L/F	5'-TCACATGTGAATGTACTAAA-3'

^a^SNPs at these three positions are genotyped in the same Pyrosequencing reaction.

^b ^Primer sits down prior to position 1711 where there is a rare polymorphism not considered in this analysis.

Sepharose-bound PCR products were captured on the probes of the Pyrosequencing Vacuum Prep Tool (Biotage, Charlottesville, VA). The beads were washed in 70% ethanol, followed by denaturation solution (0.2 M NaOH), and then washing buffer (Biotage, Charlottesville, VA) for 15 seconds each. The vacuum was released, and the probes were immersed in the PSQ HS 96-well plate containing the annealing solution, and the beads were released by gentle shaking. The plate was then incubated on a heat block at 80°C for 2 minutes and allowed to cool to room temperature prior to reading. Plates were read on a PSQ HS Pyrosequencer using PSQ HS 96A SNP reagents and analysis software version 1.2 in AQ mode. Only samples with single peak signals of at least 30 RLU (relative luminescence units) were considered suitable for allele quantification. Samples that gave "wide peak" warnings upon analysis were also rejected and the Pyrosequencing repeated.

### PCR cloning

For samples chosen for cloning, PCR products were generated using nonbiotinylated versions of the MSP-1_19 _PCR primers. These products were cloned using a PCR Cloning Plus Kit (Qiagen, Valencia, CA). Transformed cells were plated on LB plates containing 100 mg ampicillin, 80 mg X-gal, and IPTG. Clones with successful ligations were chosen by blue-white screening, followed by PCR screening with MSP-1_19 _PCR primers. Twelve clones were picked for each ligation. The nucleotide sequence of each clone was determined using Pyrosequencing and confirmed by direct sequencing.

### Standard curve generation

**Figure 1:**
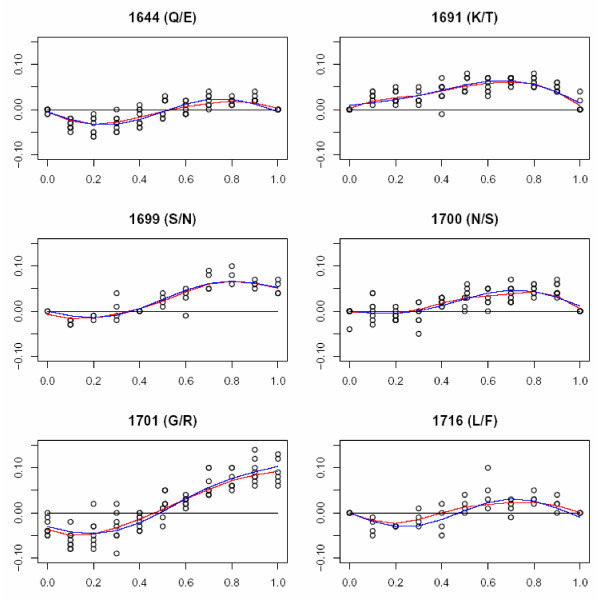
**Standard curves for each of the six single nucleotide polymorphisms in MSP-1_19_**. Graphs depict the percent deviation between expected and observed frequencies (y-axis) over a range of expected frequencies (x-axis). Circles indicate the observed frequencies, the red line indicates the smoothed data, and the blue line represents the fitted standard curve.

To account for variation in the accuracy of allele frequency determination and to standardize across the different SNPs, standard curves were generated for each of the six polymorphic positions in MSP-1_19 _(Figure 1). Experimental mixtures of MSP-1_19 _clones were created to generate standard curves and estimate the magnitude of experimental errors. Plasmids were extracted from clones using a QiaPrep Spin Miniprep kit (Qiagen, Valencia, CA). Plasmid concentrations for dilutions and mixtures were determined using a NanodropTM ND-1000 Spectrophotometer (NanoDrop Technologies, Wilmington, DE). Because no two clones differed at all six SNPs, two curves were generated: one using clones that differed at all sites except 1699 and the other using clones that differed at all sites except 1716. Plasmids were combined in ratios of 10:0, 9:1, ...1:9, 0:10. TE was added to each mixture to dilute to a final concentration of 1 ng/μl. 2 μl of each dilution was used in PCR as described above, and products underwent Pyrosequencing. To generate standard curves for each of the SNPs, the deviations between the expected and observed allele frequencies (i.e. the errors) were plotted, and a function, S_i_, was chosen to correct these errors. Standard curves were chosen from the family of curves given by the five-parameter function of the allele frequency, p: a + bp + cp^2 ^- dsin(2gπp). Backwards fitting was used to find the most parsimonious function S_i _for each of the SNPs (Table 2). Thus, given a set of measured frequencies p_i_, the best estimate of the actual frequencies is S_i_(p_i_).

**Table 2:** Standard Curves. Functions used to adjust Pyrosequencing allele frequencies for six polymorphic sites in MSP-1_19_.

**SNP Location**	Function*	**Parameter values**				
		
		**a**	**b**	**c**	**d**	**g**
1644	a - dsin(2πp)	-0.00497	-	-	0.0292	-
1691	a+bp+cp^2 ^- dsin(2πp)	0.00894	0.176	-0.170	0.0162	-
1699	bp - dsin(2πp)	-	0.0522	-	0.0261	-
1700	bp + cp^2 ^- dsin(2πp)	-	0.0966	-0.0851	0.0219	-
1701	a+cp^2 ^- dsin(2πp)	-0.0303	-	0.133	0.0223	-
1716	-dsin(2gπp)	-	-	-	0.0311	1.051

*Where p is the allele frequency at the SNP of interest

### Haplotype estimation

The haplotype-estimating algorithm uses maximum likelihood methods to determine the most probable combination of haplotypes given the allele frequencies for an infection, the haplotypes known to be circulating in the population (Table 3), and a probability distribution of the measurement errors. To estimate the distribution of measurement errors associated with each SNP (i.e. the residual errors after adjustment to the standard curve), the absolute values of the errors were assumed to be exponentially distributed. The mean residual error for each SNP, ε_i_, was calculated using the same clone mixtures that were used to generate the standard curve data. Given a putative set of haplotype frequencies, f_i_, and a set of allele frequencies, p_i_, the negative log-likelihood of f_i _is ∑_i_(|A(f_i_) - S(p_i_)|/ε_i_), where A (f_i_) indicates the allele frequencies for a putative combination of haplotypes, S(p_i_) represents observed allele frequencies adjusted to the standard curve, and ε_i _is the mean residual error for each SNP. To estimate the multiplicity of infection (MOI) for each infection, M_i_, the number of haplotypes per infection was assumed to be distributed as a conditional Poisson [19] (i.e. each infection has at least one haplotype) with a mean of 1.38 haplotypes (estimated from 296 infections from the Bandiagara malaria incidence study). Thus, the full equation for the negative log likelihood is ∑_i_[(|A(f_i_) - S(p_i_)|/ε_i_) - log(POIS(M_i_-1, 0.38))].

**Table 3:** MSP-1_19 _haplotypes observed in Bandiagara, Mali and confirmed by PCR Cloning

**Haplotype**	**Amino Acid Position**					
	
	**1644**	**1691**	**1699**	**1700**	**1701**	**1716**
	
	(E/Q)	(T/K)	(S/N)	(S/N)	(R/G)	(L/F)
1	Q	K	S	N	G	L
2	E	K	S	N	G	L
3	E	T	S	S	R	L
4	Q	K	N	N	G	L
5	E	K	S	S	R	L
6	Q	K	S	N	G	F
7	Q	T	S	S	R	L
8	E	T	S	N	G	L
9	E	T	S	S	G	L
10	E	K	N	N	G	L
11	E	K	S	N	G	F
12*	Q	K	S	S	R	L
13*	Q	K	S	S	G	L
14*	Q	T	S	S	G	L

* Not previously reported in the literature.

**Figure 2:**
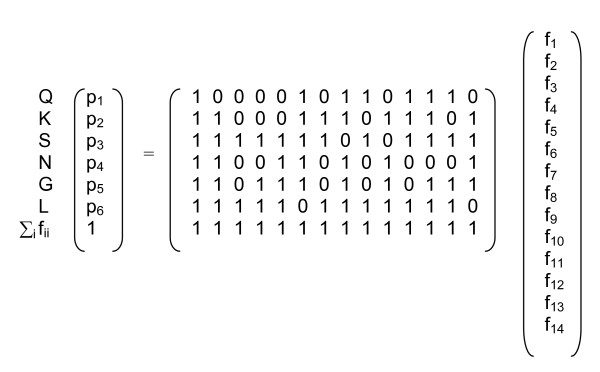
**Linear system of equations to choose starting conditions for haplotype estimation**. To address the concern of finding multiple local maxima during haplotype estimation, starting conditions were chosen by assuming the maximum number of haplotypes per infection was seven and finding all combinations of haplotypes that could explain the allele frequencies in the sample by solving a reduced linear system of equations.

Finding the combination of haplotype frequencies, f_i_, that maximizes the likelihood is hampered by the possibility of finding multiple local maxima. To address this concern, a simple optimization procedure was used with a large set of starting conditions. Starting conditions were chosen by assuming the maximum number of haplotypes per infection was seven and finding all combinations of haplotypes that could explain the allele frequencies in the sample by solving a reduced linear system of equations (Figure 2). The software was written in R (R Foundation for Statistical Computing, Vienna, Austria) and is available upon request.

### Validation of haplotype-estimating algorithm

Experimental mixtures of plasmids from MSP-1_19 _clones were made to determine the algorithm's ability to correctly estimate the haplotypes present in mixed malaria infections. Plasmids were mixed in the proportions listed in Table 4. Like the mixtures used to generate the standard curves, each mixture had a final concentration of 1 ng/μl and 2 μl were used as the template for PCR. Allele frequencies at each SNP were determined using Pyrosequencing as described above. Input for the algorithm included the standard curve adjusted allele frequencies for each sample and the list of 14 haplotypes observed in the study population.

**Table 4:** Validation of haplotype-estimating algorithm. Known haplotype frequencies present in artificial mixtures of MSP-1_19 _clones were compared to maximum likelihood estimates of haplotype frequencies generated using the algorithm.

**Mixture**	**Actual Haplotype (%)**					**Maximum Likelihood Estimate Haplotype (%)**				
		
	**1**	**2**	**3**	**4**	**5**	**1**	**2**	**3**	**4**	**5**
1	QKSNGL (70)	EKSNGL (30)				QKSNGL (73)	EKSNGL (27)			
2	QKSNGL (30)	EKSNGL (70)				QKSNGL (37)	EKSNGL (63)			
3	QKSNGL (70)	ETSNGL (30)				QKSNGL (72)	ETSNGL (28)			
4	QKSNGL (30)	ETSNGL (70)				QKSNGL (33)	ETSNGL (67)			
5	EKSSRL (70)	ETSNGL (30)				EKSSRL (70)	ETSNGL (30)			
6	EKSSRL (30)	ETSNGL (70)				EKSSRL (29)	ETSNGL (63)	**EKSNGF (07)**		
7	QKSNGF (70)	EKSSRL (30)				QKSNGF (75)	EKSSRL (25)			
8	QKSNGF (30)	EKSSRL (70)				QKSNGF (38)	EKSSRL (62)			
9	ETSSRL (35)	QKNNGL (15)	QKSNGL (50)			ETSSRL (31)	QKNNGL (14)	QKSNGL (55)		
10	ETSSRL (15)	QKNNGL (35)	QKSNGL (50)			ETSSRL (14)	QKNNGL (38)	QKSNGL (49)		
11	QKSNGL (47)	EKSNGL (20)	EKSSRL (33)			QKSNGL (50)	EKSNGL (21)	EKSSRL (29)		
12	QKSNGL (20)	EKSNGL (47)	EKSSRL (33)			QKSNGL (21)	EKSNGL (49)	EKSSRL (31)		
13	ETSSRL (23)	QKNNGL (10)	QKSNGL (33)	EKSNGL (33)		ETSSRL (21)	QKNNGL (15)	QKSNGL (31)	EKSNGL (33)	
14	ETSSRL (10)	QKNNGL (23)	QKSNGL (33)	EKSNGL (33)		ETSSRL (10)	**EKNNGL (30)**	QKSNGL (60)		
15	QKSNGL (31)	EKSNGL (13)	EKSSRL (22)	ETSSRL (33)		EKSNGL (49)	EKSSRL (18)	**QTSSRL (33)**		
16	QKSNGL (13)	EKSNGL (31)	EKSSRL (22)	ETSSRL (33)		QKSNGL (14)	**ETSNGL (33)**	EKSSRL (53)		
17	ETSSRL (15)	QKNNGL (07)	QKSNGL (22)	EKSNGL (22)	QKSNGF (33)	**QTSSRL (14)**	QKNNGL (07)	QKSNGL (47)	**EKSNGF (32)**	
18	QKSNGL (21)	EKSNGL (09)	EKSSRL (15)	ETSSRL (22)	QKSNGF (33)	**ETSNGL (17)**	EKSNGL (22)	**QKSSRL (31)**	QKSNGF (30)	

* Haplotypes indicated in bold type are not present and represent errors in haplotype estimation.

### Human subjects approval

Samples were collected under protocols reviewed and approved by Institutional Review Boards of the University of Maryland School of Medicine and the University of Mali Faculty of Medicine. Informed consent was obtained from all study participants or their guardians.

## Results

### MSP-1_19 _haplotypes in Bandiagara, Mali

A total of 20 samples underwent PCR cloning. Sequencing of the clones from these samples identified 14 unique MSP-1_19 _haplotypes circulating in the study population. The observed haplotypes are listed in Table 3. Three of these haplotypes have not been previously reported (i.e. QKSSGL, QKSSRL, and QTSSGL).

### Accuracy and precision of allele frequency determination

Several factors can affect relative peak heights generated during Pyrosequencing, including the bases flanking the polymorphic site (homopolymer formation occurs when adjacent nucleotides are identical to one of the alleles at the SNP site), increased signal from "A" alleles due to the use of dATPαS instead of dATP in the Pyrosequencing reaction, and background signal [14]. To account for these sources of variation and to standardize across the different SNPs, standard curves were generated for each of the six polymorphic positions in MSP-1_19_. Four replicates of each dilution were genotyped on two different days. There was no statistically significant difference between replicates run on different days (data not shown). The deviation between the expected and observed allele frequencies for all replicates was plotted over the range of expected frequencies (Figure 1). For each SNP, a standard curve was fitted to the data. As observed in Figure 1, the allele frequencies at site 1701 required the most adjustment, with a correction of ~10% required as the frequency of the G allele approached 100%. The other five SNPs required allele frequency corrections of <10%. Raw allele frequencies for each SNP were adjusted to the standard curve prior to haplotype estimation.

The mean residual errors for each SNP (i.e. the mean difference between adjusted individual observations and the standard curve) were 1.8%, 3.7%, 3.2%, 2.0%, 4.3%, and 1.8%, respectively for positions 1644, 1691, 1699, 1700, 1701, and 1716. These data suggest that allele frequency measurements at positions 1701 and 1691 were the least precise; however, all mean errors were less than 5%.

### Validation of haplotype-estimating algorithm

**Figure 3:**
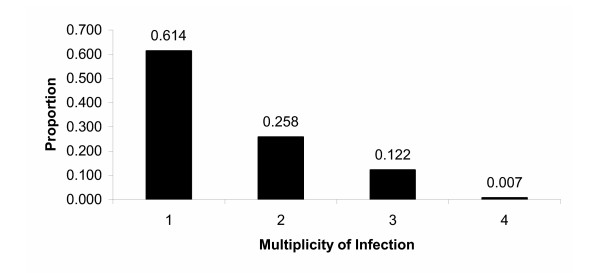
**Multiplicity of infection based on MSP-1_19_**. The number of MSP-1_19 _haplotypes observed per infection (multiplicity of infection), among 296 infections from Bandiagara, Mali, as determined by Pyrosequencing followed by haplotype estimation.

To test the algorithm's ability to correctly estimate the haplotypes present in mixed malaria infections, plasmids from MSP-1_19 _clones were used to make mixtures with known frequencies of various MSP-1_19 _haplotypes. These mixtures then underwent PCR, Pyrosequencing, and haplotype-estimation. The actual and estimated haplotypes and their frequencies are shown in Table 4. Based solely on maximum likelihood, the algorithm does very well estimating up to three haplotypes. Haplotype estimation is less accurate for four or more haplotypes. Examining the algorithm output for the higher multiplicity of infection (MOI) mixtures shows that the model yields multiple "good" answers with similar likelihoods, and consequently it is difficult to choose which "good" answer is correct (i.e. identifiability becomes a problem with high MOI infections). However, lower MOI infections make up a majority of the infections observed in Mali (Figure 3). Based on data from 296 infections from a malaria incidence study in Bandiagara, Mali, nearly 90% of infections have one or two MSP-l_19 _haplotypes (Figure 3).

## Discussion

A high-throughput method that combines allele frequency determination by Pyrosequencing with a mathematical model was developed to estimate the MSP-1_19 _haplotypes present in mixed malaria infections. After adjustment to a standard curve, Pyrosequencing yields accurate and precise estimates of the relative frequency of alleles in mixed infections. The haplotype-estimating algorithm uses maximum likelihood methods to determine the most probable combination of haplotypes given the allele frequencies for an infection and the haplotypes known to be circulating in the population, and provides accurate estimates of haplotypes present in lower multiplicity of infection (MOI) infections (≤3 types). For higher MOI infections (≥4 types), the algorithm gives statistically reasonable, but less accurate, estimates. The reduced accuracy at high MOI is primarily due to the inability of the algorithm to choose between several haplotype combinations with similar likelihoods.

Because MSP-1_19 _is highly conserved, measures of MOI based on this locus are likely to be lower than those based on more polymorphic loci (e.g. MSP-1 block 2 or MSP-2). Therefore it may be acceptable to have an algorithm with greater accuracy at lower MOI. In Mali, the vast majority of samples have low MOI (≤3 types) based on this locus. In 24 infections from six infants living in a high transmission area of western Kenya, the largest number of MSP-1_19 _haplotypes observed in an infection was two; however, the largest number of clones picked per sample was four, and it is possible that higher MOIs would have been observed had more clones been picked [11]. At a population level, with large sample sizes, the inaccurate estimation of some haplotypes in a small number of high MOI infections is not likely to be statistically relevant. When individual histories are of interest, it may be possible to fine-tune the algorithm to allow more accurate estimation of high MOI infections by using information about the haplotypes present in low MOI infections that come before and after the high MOI infection to choose the "best" answer out of several statistically "good" answers.

Identifiability problems will also increase as the number of circulating haplotypes in a population increases. Therefore, in areas of high transmission where there may be more circulating haplotypes, it may be necessary to restrict the algorithm to include the most common haplotypes. By doing so, the algorithm should be able to resolve most of the infections, and will be unable to resolve infections that contain rare haplotypes. These rare haplotypes can then be identified using other methods such as PCR cloning.

Similar expectation-maximization methods have been used to estimate haplotype frequencies in diploid human populations [20,21] and in pooled human DNA [22,23]. The expectation-maximization (EM) algorithm developed by Excoffier and Slatkin uses maximum likelihood methods to determine the most probable haplotype assignment given the observed sample genotypes and the estimated population haplotype frequencies (under the assumption of Hardy-Weinberg equilibrium). This method works best for large sample sizes, and uses several sets of starting conditions to avoid convergence on local maxima [20]. Stephens and colleagues use a Bayesian method to reconstruct haplotypes based on both the likelihood and an a priori assumption that unresolved haplotypes tend to be similar to known haplotypes [21]. The EM algorithm has recently been applied toward resolving haplotypes in pooled human DNA samples [22,23]. Similar to the algorithm described in this study, haplotype estimation in pooled human DNA samples is most accurate when the pool consists of fewer individuals. Ito et al. achieved the most accurate estimates with pools containing fewer than four individuals [22], while Quade et al. achieved accurate estimates for up to ten pooled samples (using only two alleles at two loci) [23]. These studies indicate that lack of identifiablity in samples with larger numbers of haplotypes is a common limitation of these types of algorithms.

The accuracy of Pyrosequencing allele quantification can be affected by several factors including having an "A" allele in the SNP and having flanking bases identical to one or the other alternative alleles in the SNP (i.e. homopolymer formation). Given the A/T rich genome of Plasmodium, four out of six SNPs in MSP-1_19 _contain an "A" allele. In addition, five of the six SNPs in 19 kDa form homopolymers with flanking alleles. Therefore, it is important to adjust the allele frequencies to a standard curve to improve accuracy. However, since allele frequencies of replicate runs of the same sample on different days did not differ significantly, one standard curve can be used to adjust all the data (as opposed to generating a curve every day the assay is run).

Several methods have been used to determine allele frequencies in mixed malaria infections including PCR cloning [11,24], real-time quantitative PCR (RTQ-PCR) [25], and proportional sequencing [26]. All of these methods, including Pyrosequencing, have advantages and disadvantages. PCR cloning gives definitive haplotypes; however, it is the most time-consuming and expensive of the methods, which significantly limits the number of samples that can be feasibly analyzed using this method. In addition, because *Plasmodium *often uses codons different than those used by the competent bacteria used in cloning, not all sequences can be cloned efficiently. RTQ-PCR is a more sensitive method than Pyrosequencing at detecting very low frequency alleles (<5%); however, it has a lower throughput and requires more optimization than Pyrosequencing. Like RTQ-PCR, Pyrosequencing assays are designed to detect known polymorphisms. Methods that rely on sequencing an entire region or gene of interest (e.g. PCR cloning) are better for detecting new SNPs. Proportional sequencing is a method that estimates allele frequencies in mixed infections by measuring the peak heights in direct sequencing electropherograms [26]. While this method has similar applications and accuracy as Pyrosequencing, it is more expensive and has a lower throughput [26]. Because Pyrosequencing sequences short stretches of nucleotides (10–20 bp), for certain very polymorphic loci (e.g. domain I of *P. falciparum *apical membrane antigen-1, another vaccine candidate antigen), it is not possible to set down a sequencing primer every 20 bp. In this instance, proportional sequencing may be more appropriate. If MSP-1_19 _haplotypes are of interest, allele frequencies from any of these methods can be used with the haplotype-estimating algorithm described here.

The cost of equipment for Pyrosequencing is similar to that for standard DNA sequencing, which is now done in several sub-Saharan African countries, including Mali. Pyrosequencing may be suitable for other applications such as typing known single nucleotide polymorphisms in parasite genes that serve as molecular markers for drug resistant malaria.

## Conclusion

In conclusion, Pyrosequencing is a technique that allows reliable quantification of alleles in mixed malaria infections. It is fast, relatively inexpensive, and can be used to genotype polymorphisms of interest in many important *Plasmodium *genes such as those responsible for drug resistance, immunity, and virulence. In this study, Pyrosequencing was adapted to measure the frequency of alleles in an erythrocytic vaccine candidate antigen MSP-1_19 _and combined with a haplotype-estimating algorithm to estimate the frequency of MSP-1_19_haplotypes in infected individuals. This method is being used to understand the natural dynamics of MSP-1_19_, at both population and individual levels, at a malaria vaccine-testing site in Bandiagara, Mali, and can be used to monitor populations during large-scale vaccine trials to determine allele-specific vaccine efficacy.

## Authors' contributions

SLT conceived of the molecular aspects of the study, performed the laboratory work, and worked in collaboration with DLS to develop the haplotype-estimating algorithm. DLS also helped draft the manuscript. OCS conceived of the haplotype-estimating algorithm. DC, MAT, and OKD participated in the conception, design, and conduct of the malaria incidence and case-control studies at the Bandiagara, Mali field site. CVP participated in the conception, design, and coordination of both the molecular and field studies, and helped draft the manuscript.
